# Transferrin Receptor 2 Dependent Alterations of Brain Iron Metabolism Affect Anxiety Circuits in the Mouse

**DOI:** 10.1038/srep30725

**Published:** 2016-08-01

**Authors:** Rosa Maria Pellegrino, Enrica Boda, Francesca Montarolo, Martina Boero, Mariarosa Mezzanotte, Giuseppe Saglio, Annalisa Buffo, Antonella Roetto

**Affiliations:** 1 Department of Clinical and Biological Sciences, University of Torino, Turin, Italy; 2 AOU San Luigi Regione Gonzole 10043 Orbassano Turin, Italy; 3 Department of Neuroscience Rita Levi-Montalcini, University of Torino, Turin, Italy; 4 Neuroscience Institute Cavalieri Ottolenghi Regione Gonzole 10043 Orbassano Turin, Italy

## Abstract

The Transferrin Receptor 2 (Tfr2) modulates systemic iron metabolism through the regulation of iron regulator Hepcidin (Hepc) and *Tfr2* inactivation causes systemic iron overload. Based on data demonstrating *Tfr2* expression in brain, we analysed *Tfr2*-KO mice in order to examine the molecular, histological and behavioural consequences of *Tfr2* silencing in this tissue. *Tfr2* abrogation caused an accumulation of iron in specific districts in the nervous tissue that was not accompanied by a brain Hepc response. Moreover, *Tfr2-*KO mice presented a selective overactivation of neurons in the limbic circuit and the emergence of an anxious-like behaviour. Furthermore, microglial cells showed a particular sensitivity to iron perturbation. We conclude that Tfr2 is a key regulator of brain iron homeostasis and propose a role for Tfr2 alpha in the regulation of anxiety circuits.

Body iron amount and availability is finely regulated by Hepcidin (Hepc), a peptide produced mainly by the liver, that acts on the cellular iron exporter Ferroportin 1 (Fpn1), causing its degradation and decreasing *de facto* the amount of serum iron^1^. A complex protein network is involved in hepatic Hepc regulation according to the body iron needs, causing Hepc decrease in iron demand conditions (anaemia, hypoxia, ineffective erythropoiesis) and, on the contrary, a Hepc increase when a sufficient iron amount is present in the body or during inflammatory processes^2^. Dysfunction of most Hepc regulatory proteins is responsible of hereditary disorders of iron metabolism^3^,^4^. Among these regulators, Transferrin receptor 2 (Tfr2) is codified by a gene whose mutations are responsible for a rare form of hereditary haemochromatosis named HFE3^5^,^6^. The *TFR2* gene is transcribed in two main isoforms, Tfr2 alpha and Tfr2 beta. Tfr2 alpha is highly produced in the liver and works as an iron sensor that regulates serum Hepc level. Accordingly, *Tfr2* targeted animals show iron overload due to an inappropriately low level of Hepc^7^,^8^,^9^. The Tfr2 beta isoform, instead, appears to play a specific role in the regulation of iron export from reticulo-endothelial cells^9^.

In the central nervous system (CNS), iron levels need to be tightly controlled to appropriately regulate key functions such as neurotransmission and myelination as well as neural cell division^10^. Iron overload in defined CNS areas associates with neurodegeneration in Parkinson’s and Alzheimer’s diseases^11^,^12^,^13^, suggesting a role for iron overload in circuit malfunctioning and damage. Furthermore, studies in animal models in which iron amount was experimentally increased show an alteration of the main iron-related proteins Ferritin (Ft) and Transferrin Receptor 1 (Tfr1) in the brain^14^,^15^,^16^, revealing perturbation of iron brain homeostasis.

It is generally accepted that iron enters neurons^10^, microglial^17^,^18^ and choroid plexi cells^19^ bound to Tfr1 and it has been shown that the iron hormone Hepc is expressed in the brain^20^,^21^,^22^,^23^, similar to other Hepc regulatory proteins^10^. Yet, it is still unclear how iron levels and localization are regulated in cerebral compartments. Similarly, it remains to be fully understood whether the iron protein regulatory network in the CNS is the same operating in the rest of the body and how systemic and cerebral iron regulation are interconnected.

Similar to other Hepc regulatory proteins, *Tfr2* gene expression has been shown in total brain extracts^23^, in defined cellular compartments of specific neuronal subtypes^24^ and in brain tumor cell lines^23^,^25^. Furthermore, a transcriptome study on *Tfr2* null mice revealed that several genes involved in the control of neuronal functions are abnormally transcribed^16^.

In this study we aimed to clarify *Tfr2* functions in the brain by examining a mouse model in which both *Tfr2* isoforms are inactivated (*Tfr2-*KO)^9^. To distinguish the effects of *Tfr2* abrogation from those due to *Tfr2*-independent iron load modifications, we also examined WT sib pairs subjected to an iron enriched diet (IED) and WT or Tfr2-KO mice upon an iron deficient diet (IDD).

Results show that *Tfr2* silencing determines an increased brain iron availability that associates with anxious-like behaviours.

## Materials and Methods

### Animals

Ten weeks old *Tfr2*-KO male mice^9^ maintained on 129 X 1/svJ strain were analyzed and compared to wild type (WT) sex and age matched sib pairs. Animal housing and all the experimental procedures were performed in accordance with European (Official Journal of the European Union L276 del 20/10/2010, Vol. 53, p. 33–80) and National Legislation (Gazzetta Ufficiale n° 61 del 14/03/2014, p. 2–68) for the protection of animals used for scientific purposes. Groups of 4–5 mice were housed in transparent conventional polycarbonate cages (Tecnoplast, Buggirate, Italy) provided with sawdust bedding, boxes/tunnels hideout as environmental enrichment and striped paper as nesting material. Food and water were provided ad libitum; environmental conditions were 12 h/12 h light/dark cycle, room temperature 21 °C ± 1 °C and room humidity 55% ± 5%. Experimental procedure was preventively approved by the Ethical Committee of the University of Turin.

### Experimental conditions

*Tfr2*-KO mice were fed with a standard diet (SD) (GLOBAL DIET 2018, Mucedola SrL, Italy, 0, 2 g iron/Kg food). Age and sex matched animals in SD were used as WT controls. Subgroups of mice were further kept on:

*a) iron deficient diet (IDD)* to induce anaemia in WT mice and to decrease iron amount in Tfr2-KO mice, by feeding mice with a purified diet without added iron (Mucedola SrL, Italy) starting from weaning until sacrifice (8 weeks of treatment);

*b) iron enriched diet (IED)* to trigger secondary iron overload in WT animals by feeding them with a 2% iron enriched standard diet from weaning until sacrifice (8 weeks of treatment).

IED was preferred to parenteral iron injection because it resembles iron overload occurring in chronic haemochromatosis. Furthermore, since it has been demonstrated that an iron enriched diet for a short period of time does not cause changes in iron regulating proteins^26^, we decided to submit animals to 8 weeks of IED. On the other hand, IDD treatment was chosen to avoid the side-effects of acute iron deprivation obtained by extensive phlebotomies that, by causing inflammation, influence the Hepc pathway^27^,^28^.

At the end of the experiments, mice were given an anaesthetic overdose (see below) and sacrificed. Blood, brain and liver were collected for subsequent analysis.

For behavioural analyses, 8 WT and 9 *Tfr2-*KO naïve mice in SD were tested in the *Morris water maze* and elevated plus maze (*EPM) tests*. Subsequently, 19 WT and 13 *Tfr2-*KO naïve mice in SD, 12 WT and 17 *Tfr2-*KO naïve mice in IDD and 5 WT naïve mice in IED were tested in EPM.

A subset of *Tfr2*-KO and WT mice was transcardially perfused with 0.12 M phosphate buffer (PB) pH 7.2–7.4 (50 ml, 15 min) to remove blood from brain tissue before Western Blot analysis and the measurement of brain iron content (see below). Perfusions were carried out under deep general anaesthesia (ketamine, 100 mg/kg; Ketavet, Bayern, Leverkusen, Germany; xylazine, 5 mg/kg; Rompun; Bayer, Milan, Italy).

### Molecular biology analyses

Frozen dissected regions or total brains from WT mice were utilized for quantitative PCR while only total brain of Tfr2-KO and WT animals was used for Western Blot analysis.

#### Real time quantitative PCR analysis

For reverse transcription, 1 μg of total RNA, 25 μM random hexamers, and 100 U of reverse transcriptase (Applied Biosystems) were used. Gene expression levels were measured by real-time quantitative PCR in a CFX96 Real-Time System (BIO-RAD). For Tfr2 alpha, Tfr2 beta and BDNF, SYBR Green PCR technology (EVAGreen, BIO-RAD, Italy) was used utilising specific primers whose sequences are reported in Supplemental Informations. *Gus* (β-glucuronidase) gene was utilized as housekeeping control^9^,^29^. The results were analysed using the ΔΔ_Ct_ method^30^. All analyses were carried out in triplicate; results showing a discrepancy greater than one cycle threshold in one of the wells were excluded.

#### Western Blot analysis

WB experiments were done with at least 6 animals for each experimental group. Fifty μg of total brain lysates were separated on an 8–15% SDS polyacrylamide gel and immunoblotted according to standard protocols. Antibodies against the following proteins were used: Transferrin (Tf) (F-8), Tfr1 (CD71 H-300), Divalent Metal Transporter1 (DMT1) (H-108), Fpn1 (G-16) and β-Actin (C-4) (*Santa Cruz Biotechnology*); Tfr2 alpha and Hepc (Alpha Diagnostic International). Antibodies against the two Ferritins (Ft-H and Ft-L) were kindly provided by Sonia Levi, University of Vita Salute, Milan, Italy. Data from WB quantification (Image Lab Software, BIO-RAD, Italy) were normalized on levels of β-Actin bands and expressed as fold increase relative to the mean value obtained from WT mice.

### Liver and brain iron content

Iron concentration on livers (Liver Iron Content, LIC) and brains (Brain Iron Content, BIC) freshly dissected was assessed according to standard procedures^9^ using at least 20 mg of dried total tissue. For histological assessment of non-heme iron deposition, brain slices of perfused animals were stained with DAB-enhanced Prussian blue Perls’ staining^31^.

### Histological and immunofluorescence procedures

For histological analyses experimental animals were perfused with 4% paraformaldehyde in PB. Brains were removed and post-fixed for 24 h at 4 °C, cryoprotected in 30% sucrose in 0.12 M phosphate buffer and processed according to standard protocols^32^. Brains were cut in 30 μm thick coronal sections collected in PBS and then stained to detect the expression of different antigens: Glial fibrillary acidic protein (GFAP) (1:1000, Dakopatts); Iba1 (1:1000, Wako); cFos (1:1000, Santa Cruz Biotechnology); Zif-268 (1:1000, Santa Cruz Biotechnology); vGlut1 (1:1500, Synaptic System); vGlut2 (1:1500, Synaptic System); Tfr2 alpha (1:500, Alpha Diagnostics); hepcidin/pro-hepcidin (1:1000 with amplification with tyramide kit, see below, Alpha Diagnostics). Incubation with primary antibodies was made overnight at 4 °C in PBS with 0.5% Triton-X 100. The sections were then exposed for 2 h at room temperature (RT) to secondary Cy3- (Jackson ImmunoResearch Laboratories, West Grove, PA) and Alexafluor- (Molecular Probes Inc, Eugene Oregon) conjugated antibodies^33^. 4,6-diamidino-2-phenylindole (DAPI, Fluka, Milan, Italy) was used to counterstain cell nuclei. After processing, sections were mounted on microscope slides with Tris-glycerol supplemented with 10% Mowiol (Calbiochem, LaJolla, CA). For colabelling of primary antibodies developed in the same species, the high sensitivity tyramide signal amplification kit (Perkin Elmer, Monza, Italy) was utilized according to the manufacturer’s instruction^33^. Myelin Gallyas staining was performed as documented^34^.

### Image Processing and Data Analysis

Histological specimens were examined using an E-800 Nikon microscope (Nikon, Melville, NY) connected to a colour CCD Camera and a Leica TCS SP5 (Leica Microsystems, Wetzlar, Germany) confocal microscope. Adobe Photoshop 6.0 (Adobe Systems, San Jose, CA) was used to adjust image contrast and assemble the final plates. Quantitative evaluations (densitometry of staining intensities, cell densities) were performed on confocal images followed by Neurolucida- (MicroBrightfield, Colchester, VT) and ImageJ- (Research Service Branch, National Institutes of Health, Bethesda, MD; available at: http://rsb.info.nih.gov/ij/) based analyses. For the analysis of microglia in the cerebral cortex, we routinely scanned the entire cortical grey matter included in slices from Bregma 1.10 mm to Bregma-2.00 mm. Analyses were performed on slices from Bregma-1.50 mm to Bregma-2.00 mm for the dorsal hippocampus; from Bregma-2.50 mm to Bregma-3.00 mm for the ventral hippocampus; from Bregma1.50 mm to Bregma 2.20 mm for the medial prefrontal cortex (mPFC); from Bregma-1.20 mm to Bregma-1.60 mm for the basolateral and central amygdala (BLA and CeA); from Bregma-0.70 mm to Bregma-1.00 mm for the hypothalamic periventricular nucleus (PVN). When the high density of cFos/Zif-268-positive cells impaired the easy recognition of individual nuclei (i.e., in *Cornu Ammonis* 1, CA1 and medial prefrontal Cortex, mPFC), the mean cFos/Zif-268 staining intensity (with background subtraction) over the whole area of the region of interest was evaluated. Measurements derived from at least 3 sections per animal. At least three animals were analysed for each experimental condition.

### Behavioural tests

The *Morris water maze*^35^ and EPM^36^ tests were performed to evaluate learning and anxious-like behaviours, respectively. Data were recorded automatically from the digitized image by using a computerized video tracking software. Details about the procedures can be found in Supplemental Information.

### Serum Collection and Analysis

Blood samples were collected, centrifuged, and serum was frozen at −20 °C until analysis. Serum was assayed for corticosterone levels by using commercially available kits (Corticosterone 3H RIA, MP Biomedicals, Italy). All the blood samples were collected at the same time in the morning to minimize the physiological variations.

### Statistical analysis

Statistical analyses were carried out by GraphPad Prism (San Diego California, USA) or SPSS software packages (Bangalore, India, www.spss.co.in). In most cases we used Unpaired t-test or one-way ANOVA followed by Bonferroni’s post hoc analysis. As regards behavioural data, repeated-measures two-way ANOVA followed by Bonferroni’s post hoc analysis was performed to evaluate *Morris water maze* performances during days. Mann-Whitney U test was assessed to evaluate the statistical significance for Accuracy Ratio (AR) and path length in *Morris water maze* test. One-way ANOVA followed by Bonferroni’s post hoc analysis was used to evaluate the EPM performances. In all instances, P < 0.05 was considered as statistically significant. Data were expressed as averages ± standard error of the mean. Only statistically significant results vs WT and vs *Tfr2*-KO were shown in the figures while all statistically significant P and F values as well as outputs of post hoc analyses were included in Table 1S. Statistically not-significant results were omitted.

## Results

### Tfr2 alpha expression in anxiety and stress-related circuits

**Figure 1:**
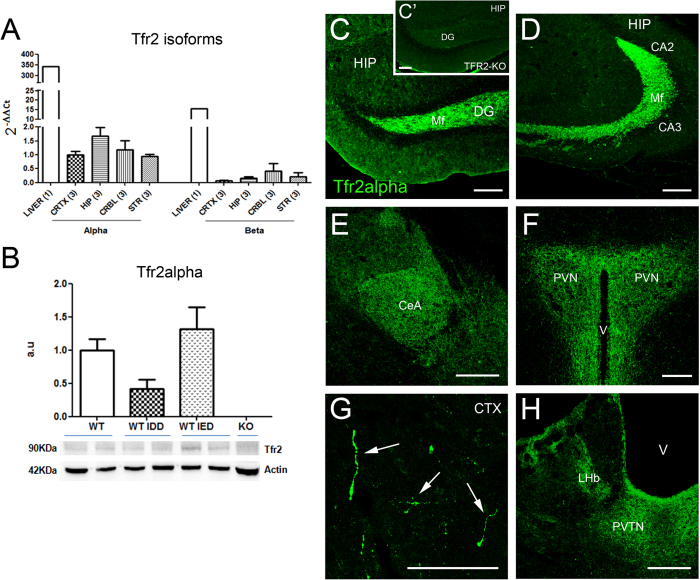
Tfr2 alpha expression in the adult telencephalon. (**A**) Tfr2 alpha and beta isoforms transcription pattern in major brain compartments of WT SD mice. CRTX, cortex; HIP, hippocampus; CRBL, cerebellum; STR, striatum; transcription amount of both Tfr2 isoforms in the liver was used as comparison. Number in parenthesis under each column indicates the number of animals compartments analysed. The expression level of the two Tfr2 isoforms was normalized to levels of GUS housekeeping gene as described in the MM section. In order to perform a double comparison of the same genes in different brain compartments and of the two Tfr2 isoforms in the same compartment we normalized all data on the value of Tfr2 alpha in the cerebral cortex (CRTX). (**B**) Western blot analysis and quantification of brain Tfr2 alpha protein. Results are shown as averages ± standard error of the mean of 3 independent experiments. All the statistically significant results are reported in Table 1S. a.u. arbitrary unit; SD standard diet, IDD iron deficient diet; IED iron enriched diet. (**C**–**H**) Immunofluorescence localization of Tfr2 alpha in brain. In WT brains Tfr2 alpha positivity is found in fibers of the dentate gyrus of the hippocampus and their terminal region in the CA3/CA2 areas (**D**). A dense dotted staining is also detected in the CeA (**E**), PVN (**F**), LHb (**H**) and PVN (**H**) grey matter nuclei. Sparse axon fibers are labelled for Tfr2 alpha in the cerebral cortex (**G**). Absence of staining in *Tfr2*-KO mice confirms the antibody specificity (C’). Scale bars: 100 μm. DG, dentate gyrus; CA, Cornus Ammonis; CeA, central nucleus of the amygdala; PVN, paraventricular nucleus of hypothalamus; CTX, neocortex (primary M1 motor cortex); LHb, lateral habenula; PVTN, paraventricular nucleus of thalamus; Mf, mossy fibers; V, ventricle.

A transcriptional analysis on WT mice major brain compartments revealed that Tfr2 alpha mRNA is expressed, although less than in the liver, in all analysed areas, and reaches the highest level in the hippocampus (Fig. 1A).

Conversely, real time analysis, performed with primers of similar efficiency, showed a low and homogeneous level of Tfr2 beta mRNA expression in the same brain areas that, also in this case, was lower compared to the liver (Fig. 1A). Based on these data, we concluded that Tfr2 alpha is the main Tfr2 isoform in the brain and consequently we focused on Tfr2 alpha for the next experiments.

Tfr2 alpha protein in total brain extracts showed a trend to decrease in WT IDD mice compared to WT mice, while it did not vary in WT IED animals (Fig. 1B). These data are in line with the role of Tfr2 as an iron sensor contributing to iron homeostasis and with previous data demonstrating that Tfr2 protein is stabilized on plasma membranes by iron loaded Transferrin (Fe-Tf)^37^.

Immunofluorescence labelling with an anti-Tfr2 alpha specific antibody showed elongated thin structures occurring either as tightly associated fascicles or isolated fibers (Fig. 1C,G). In grey matter nuclei, a dense punctate staining was occasionally seen (Fig. 1E,F,H). Both patterns are consistent with labelling of neurites or fiber tracts. Interestingly, anti-Tfr2 alpha labelling was prominent in brain circuits controlling anxiety and stress^38^,^39^ including the hippocampus (where Mossy fibers, Mf; were strongly stained, Fig. 1C,D), the amygdala (namely the central nucleus, CeA; Fig. 1E) and the hypothalamic paraventricular nucleus (PVN; Fig. 1F). Scattered Tfr2 alpha-positive (+) fiber-like structures were also found in the in the cerebral cortex (Fig. 1G) and in thalamic paraventricular and habenular nuclei (Fig. 1H). Absence of labelling in *Tfr2*-KO mouse brain testified the specificity of anti-Tfr2 alpha staining (Fig. 1C’).

### Increased iron amount in Tfr2-KO brain

All mice were examined at 10 weeks of age, when tissue alterations have been observed in other mouse models of iron loading^40^,^41^. *Tfr2*-KO showed a significant increase in total brain iron amount compared to WT mice (Table 1). However, this increase was attributable to circulating iron, since brains of Tfr2-KO mice in which blood was removed though tissue perfusion, have a BIC (Brain Iron Content) similar to WT (Table 1). Interestingly, aged-matched WT IED mice, despite being iron overloaded in the liver (Table 1) and in the serum (Transferrin saturation: 49,8 ± 4.45% vs 26 ± 4.95% P < 0,05), did not display evidence of global iron increase in the brain. In both *Tfr2*-KO and WT mice in IDD, BIC decreased below controls levels (Table 1).

**Figure 2:**
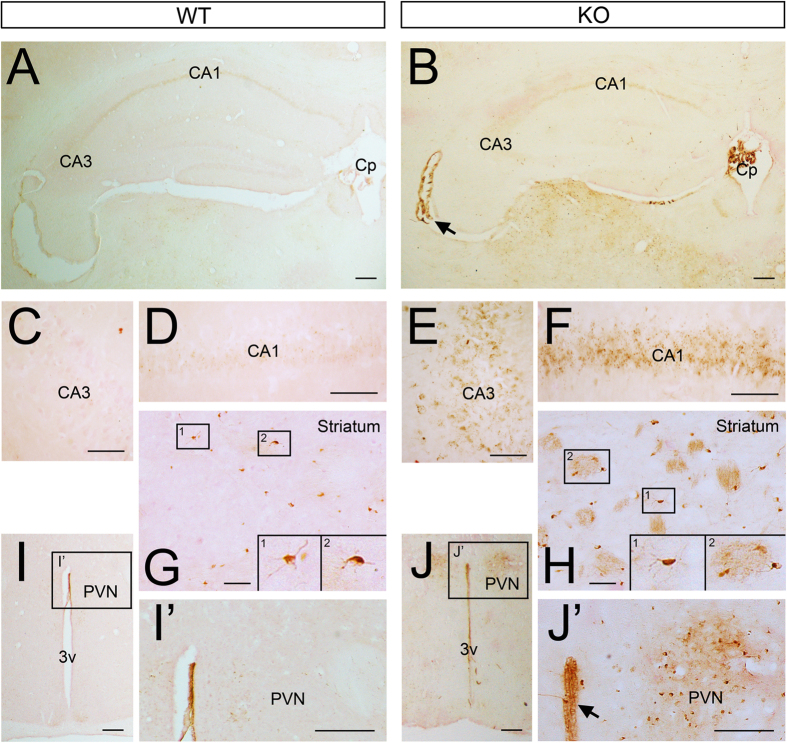
Iron accumulation in *Tfr2*-KO mouse brain. Iron in sections of the brain of WT and *Tfr2*-KO mice (**A**,**B**) DAB-enhanced Prussian Blue staining revealed iron accumulation in the choroid plexi (Cp) and ependyma (arrows) of *Tfr2*-KO mice (**B**,J’) compared to WT controls (**A**,I’). Higher magnification analysis also showed increased density of brown precipitates in the CA1 and CA3 of the mutant hippocampus (**E**,**F**), striatum (**H**) and periventricular nucleus (**J**,J’) compared to WT tissues (**C**–I’). (**G**,**H**,I’,J’). Iron labelling also decorates small glial cells that appeared more frequent in*Tfr2*-KO mice. This pattern was confirmed on 3 *Tfr2-*KO and WT mice. Scale bars: (**A**,**B**,I–J’): 100 um; (**B–G**): 50 um. CA1, Cornus Ammonis 1, CA3, Cornus Ammonis 3, Cp, choroid plexus, PVN, paraventricular nucleus, 3v, third ventricle.

To assess possible localised changes in iron levels or distribution, we performed histochemical analysis in PFA perfused brains by DAB-enhanced Prussian blue Perls’ staining. *Tfr2*-KO brains sections showed an increased number of brown positive precipitates compared to control mice (Fig. 2) in defined parenchymal regions such as the hippocampal CA1 and CA3 regions (Fig. 2C–F), the PVN (Fig. 2I–J’) and the striatal white matter (Fig. 2G,H). We also observed DAB-positive small cells, mostly resembling microglia (insets in 2G and H), that were more frequently detected in *Tfr2*-KO brains (Fig. 2A,B,I–J’). Increased iron was also highlighted by intense staining of both choroid plexi and ependyma in mutant mice (Fig. 2A,B,I,J). These results show that iron accumulates in the nervous tissue when Tfr2 is abrogated.

### Brain Hepc levels in iron overloaded Tfr2-KO mice

**Figure 3:**
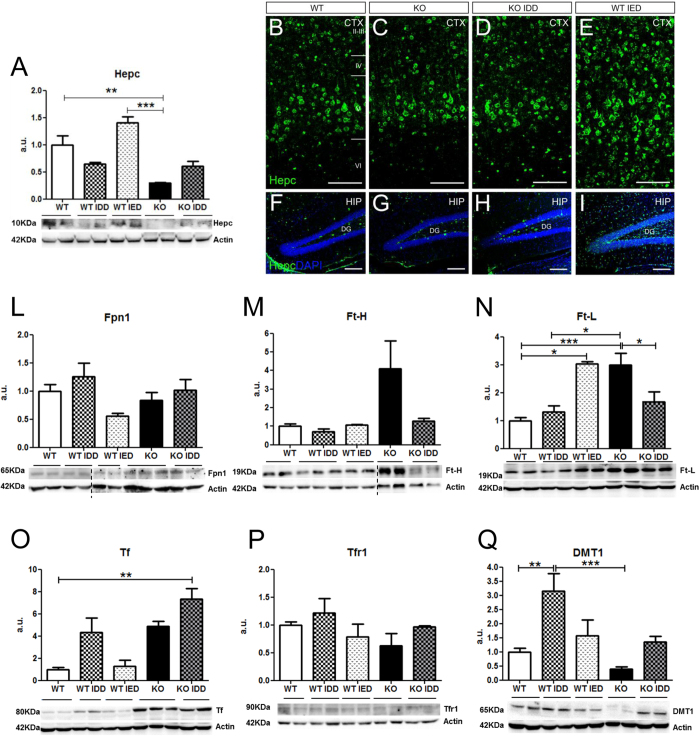
Hepcidin production and iron transport/storage proteins quantification in the brain. (**A**) Western blot analysis and quantification of Hepcidin (Hepc) production changes. (**B**–**I**) Immunofluorescence with Anti-Hepc antibody in the neocortex. Scale bars: 100 μm. Western blot analysis and quantification of (**L**) Ferroportin (Fpn1) (**M**) Ferritin H (Ft-H) (**N**) Ferritin L (Ft-L) (**O**) Transferrin (Tf) (**P**) Transferrin receptor 1 (Tfr1) (**Q**) DMT1. Results are shown as averages ± standard error of the mean. Symbols refer to a statistically significant difference: *P < 0.05, **P < 0.01, ***P < 0.001. For reasons of clarity, only statistically significant results vs WT and Tfr2 KO are shown in the figure. All the other statistically significant results are reported in Table 1S. Vertical dotted lines indicate images taken from different gels. WT, wild type; KO, *Tfr2*-KO; IDD, iron deficient diet; IED, iron enriched diet; a.u., arbitrary unit; DG, dentate gyrus; HIP, hippocampus; CTX, neocortex (primary M1 motor cortex).

Since Tfr2 is a regulator of Hepc production^2^ in the liver, we asked whether its deletion also affected Hepc amount in the brain. In agreement with its role of negative regulator of iron availability, in liver and brain of WT mice Hepc production changed according to the different systemic iron amount: it decreased in animals on IDD and increased in animals on IED (Table 1, Fig. 3A). On the contrary, in Tfr2-KO mice, despite increased systemic and circulating iron, cerebral Hepc was significantly lower than in WT mice (Fig. 3A). Furthermore, while liver Hepc transcription significantly decreased in *Tfr2*-KO IDD mice (Table 1) brain Hepc transcription increased in consequence of IDD, reaching WT IDD levels (Fig. 3A) and suggesting a deregulated expression of Hepc in the KO brain. In order to distinguish the contribution of the circulating protein to the Hepc level found in *Tfr2*-KO brain, Hepc quantification was repeated in brains of perfused animals. Despite iron accumulation in KO brains, there is no obvious dysregulation of brain-derived Hepc. These data are consistent with lack of overexpression of Hepc in perfused *Tfr2*-KO brain (Fig. 1S).

To further corroborate these data with an independent approach, we examined anti-Hepc immunostaining on brain slices. Consistently with previous data^42^, Hepc positive cells in the cerebral cortex and hippocampus mainly displayed neuronal morphologies (Fig. 3B,C,F,G). In these areas no relevant differences were observed in the Hepc expression pattern or in the densities of positive cells of WT and *Tfr2*-KO mice, although the latter seems to have a slight decrease in overall staining. Conversely, and in line with systemic Hepc regulation^2^, WT IED mice had a marked increase in the number of Hepc + cells in the cortex and in the hippocampal dentate gyrus (DG) (Fig. 3E–I). Surprisingly, and on the contrary to what occurs in the liver, where Hepc transcription significantly decreases (Table 1), the pattern and the density of brain Hepc + cells in *Tfr2*-KO IDD mice remains comparable to WT (Fig. 3B–D,F–H), confirming the result of WB analysis.

### Brain iron regulatory proteins are altered in Tfr2-KO mice

Transcriptional analysis of main Hepc regulatory genes, *Hfe* and *Hjv*^2^ did not reveal significant variations in the brain of *Tfr2*-KO mice compared to WT animals (not shown). Total brain production of the Hepc target protein Fpn1^43^, resulted to be modulated in the different WT experimental groups with an opposite trend compared to cerebral Hepc amount, even if results did not reach statistical significance (Fig. 3L). Inverse relationship between the two proteins in WT animals is further evidenced by a very good fitting in regression analysis, while an apparent opposite trend, despite not statistically different from the WT pattern, could be observed in *Tfr2-*KO mice (Fig. 2S).

To molecularly analyse the increased amount of intracellular iron in the brain parenchyma, we evaluated the three main iron proteins responsible of cellular iron storage (Ft subunits H and L) and transport (Tf). They all resulted modulated. While in *Tfr2*-KO mice brain both Ft-H and Ft-L were higher, in WT IED mice only Ft-L was significantly increased (Fig. 3M,N). Iron transport protein Tf was incremented in IDD mice, as expected on the basis of its capability to supply with iron tissues in which iron amount is decreased^44^,^45^ (Table 1). Surprisingly, also *Tfr2*-KO mice presented an overall higher Tf amount, while this was not true for WT IED mice (Fig. 3O).

Again to verify that these proteins amount was not due to blood presence in non-perfused brains, Fts and Tf were analysed in brain of perfused animals. Blood removal in *Tfr2*-KO brains seems to cause a decrease of the three proteins amount. While an overall increase for Fts was confirmed, Tf displayed levels similar to WT brains, indicating a major role of circulating Tf in measurements in non-perfused brains (Fig. 1S).

As regards the main proteins responsible of cellular iron import, no significant variations were found for Tfr1 levels in all the experimental groups (Fig. 3P). A lower amount of DMT1 protein was instead observed in *Tfr2*-KO brains compared to WT controls (Fig. 3Q). This result supports the hypothesis that the higher iron amount in *Tfr2*-KO mouse brains triggers a decrease in DMT1 protein according to the IRE/IRP pathway^45^. The same regulatory mechanism accounts for DMT1 increase in WT IDD mice compared to WT, as well as in *Tfr2*-KO IDD animals compared to *Tfr2*-KO (Fig. 3Q).

In conclusion, Tfr2 silencing associates with changes in both CNS iron import and storage proteins, in line with an altered cellular distribution and availability of the metal in the brain of these mice.

### Tfr2-KO mice exhibit increased anxiety

**Figure 4:**
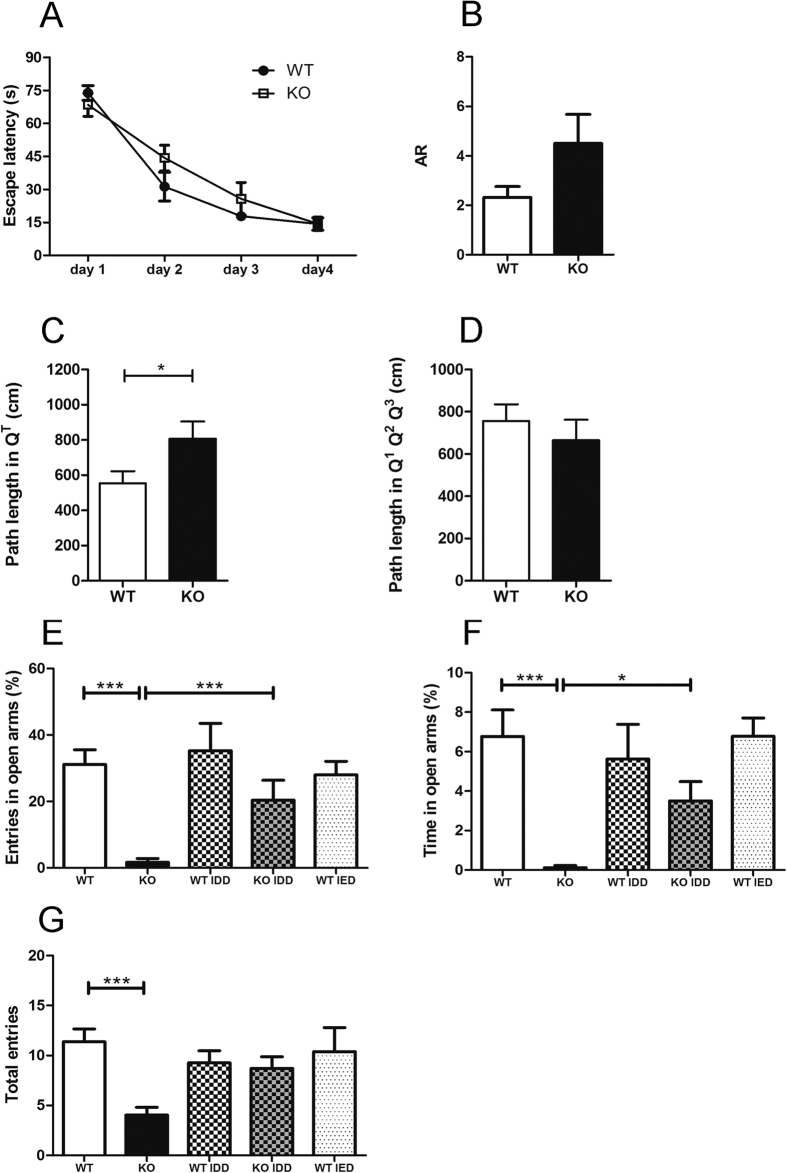
Overt anxiety-like behaviour in Tfr2-KO mice. (**A**,**B**) Performance in Morris water maze test. Both WT (n = 8) and Tfr2-KO (n = 9) mice improve their performance across days and during the probe trial without differences between genotypes. (**C**,**D**) Path length in Morris water maze test. Measures of the total distance (cm) covered by WT mice and Tfr2-KO mice after they reached the target zone in the probe trial. (**C**) Tfr2-KO mice showed longer path length in the target quadrant (Q^t^) compared to WT mice. No differences are found in quadrants outside the target zone (Q^1^, Q^2^, Q^3^). (**E–G**) Performance in EPM test. Tfr2-KO (n = 22) mice reveal anxiety levels higher than WT SD mice (n = 27). In IDD, Tfr2-KO mice (n = 17) show a rescue in anxious behaviors and perform similarly to WT mice (n = 12). AR, accuracy ratio; EPM, elevated plus maze; SD, standard diet, IDD; iron deficient diet, IED, iron enriched diet; Q_T_ target quadrant; error bars, standard error of the mean. Asterisks refer to statistically significant differences: *P < 0.05, **P < 0.01, ***P < 0.001. Statistically significant results are reported in Table 1S.

Based on high expression of Tfr2 in the hippocampus and limbic circuits, we examined learning abilities and anxiety in the *Tfr2*-KO mice by behavioural tests. In the Morris water maze test no differences were found between WT and *Tfr2*-KO mice in the initial performance (day 1) (Fig. 4A). Furthermore, both WT and *Tfr2*-KO mice were able to improve their performance across days without differences (Fig. 4A). In the probe trial, the mean accuracy ratio (AR) did not show any significant difference between WT and *Tfr2*-KO mice, although *Tfr2*-KO mice spent about 2 fold more time in the target quadrant compared to wild type mice (Fig. 4B). Also in swim velocity and distance there were no differences between WT and *Tfr2*-KO mice (mean velocity ± SE, WT = 23.1 ± 1.4 cm/s, *Tfr2*-KO = 26.4 ± 0.8 cm/s; mean travelled distance ± SE, WT = 1380 ± 83.9 cm, Tfr2-KO = 1582 ± 48.4 cm; Mann-Whitney U; P > 0.05). Thus, *Tfr2*-KO mice do not show impairments of hippocampal-related spatial memory tasks. However, the trend for a stronger preference for the probe quadrant in *Tfr2*-KO mice (Fig. 4B) led us to measure the path length after mice reached the target zone. An increased path length outside the target zone, after reaching the original location of the platform, would suggest increased flexibility in an attempt to look for a new location of the platform. On the contrary, longer path length in the target zone would suggest persistency possibly related to increased anxiety^35^. *Tfr2*-KO mice displayed a significantly longer path length in the target zone after reaching the original position occupied by the platform compared with their WT sib pairs (Fig. 4C,D).

Furthermore, to avoid confounding effects due to changes in mutants of innate preference for swimming in defined areas of the maze^46^, we calculated the distance travelled and the time spent in the centre zone of the pool versus the periphery region on the first trial of the first day, when the spatial location of the platform was completely unknown to the mice. WT and *Tfr2*-KO mice did not show significant differences in the percentages of travelled distances and time spent in the centre of the pool (mean percentage of distance travelled in centre ± SE, WT = 7.1 ± 3.4, *Tfr2*-KO = 7.1 ± 2.2; mean percentage of time spent in centre ± SE, WT = 5.4 ± 2.5 cm, *Tfr2*-KO = 5.8 ± 2.0 Mann-Whitney U; P > 0.05) thus showing that both WT and *Tfr2-*KO mice have the same innate preference for swimming in distinct areas of the maze.

Finally, we further assessed anxiety in the EPM. Notably, *Tfr2*-KO mice showed increased anxiety as expressed by a dramatically low frequency of entries in the open arms of the EPM (Fig. 4E). Consistently, they spent a little time in the open arms of the EPM (Fig. 4F) compared with WT siblings. Also, the total number of entries was reduced in *Tfr2*-KO mice (Fig. 4G). Then, we asked whether such anxious-like behaviour depends on iron levels by examining mice subjected to IDD and IED. Reductions in the frequency and time spent in open arms and in total entries were reverted to control values by IDD in *Tfr2*-KO mice (Fig. 4E,F,G). Notably, neither IDD nor IED affected the anxious behaviour of WT mice (Fig. 4E,F). Altogether, these data show that loss of Tfr2 associated with iron overload promotes the occurrence of anxious behaviours.

### Higher levels of activation of the anxiety circuitry in Tfr2-KO mice

**Figure 5:**
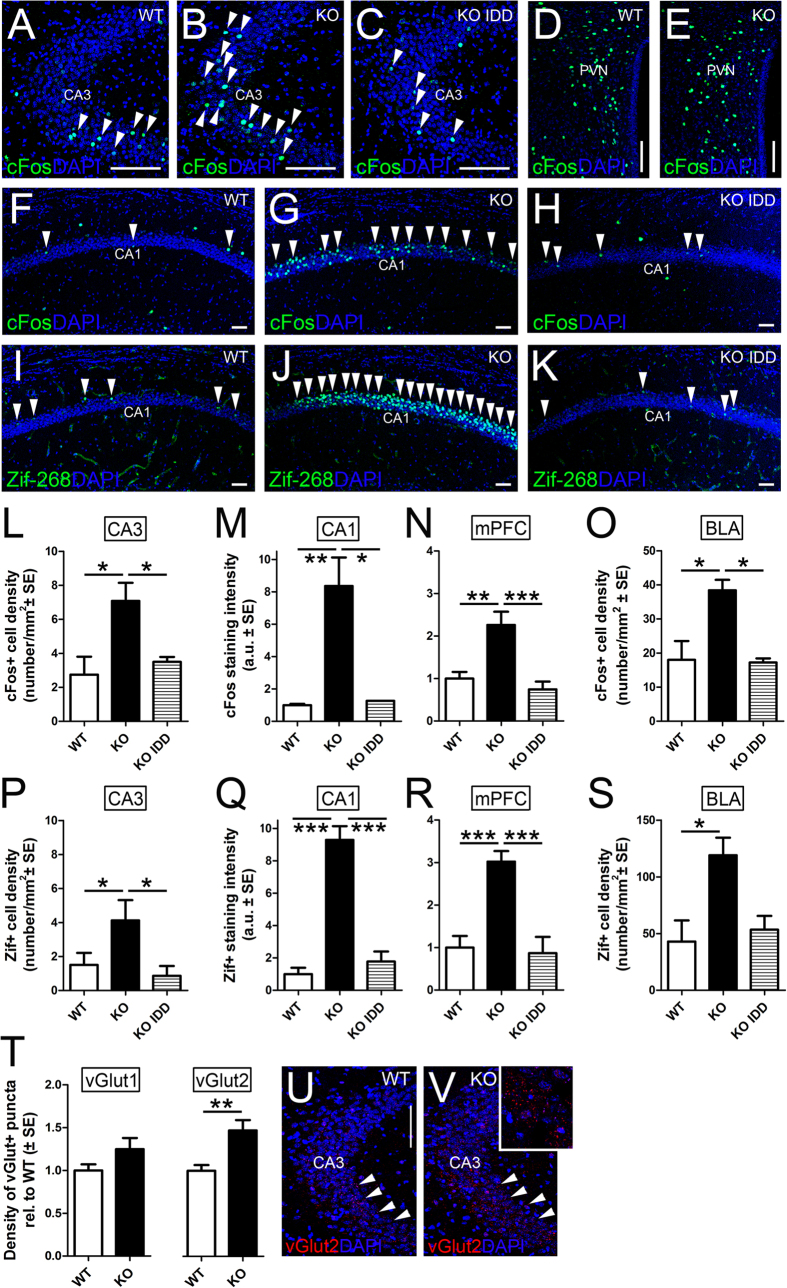
Activity-related immediate early genes in anxiety circuits. The immediate early genes cFos (**A**–**H**) and Zif-268 (**I**–**K**) are upregulated in neurons of the CA1 (**F**,**G**) and CA3 (**A**,**B**) areas of *Tfr2*-KO mice compared to WT brains, while no increase in positive cells occurs in the PVN (**D**,**E**). Quantifications of the number of positive nuclei over the area of the corresponding layers show that cFos+ or Zif-268+ cells significantly increased in mutant mice in standard conditions while they return to control levels in mutant fed with IDD diet (**H**,**K**,**L**,**M**,**P**,**Q**). This very same trend is found in the mPFC and BLA (**N**,**O**,**R**,**S**). (**T**–**V**) Quantifications of glutamatergic terminals in CA3 (red in (**U**,**V**)) show that vGlut2+ puncta are higher in number in *Tfr2*-KO mice, while vGlut1+ ones do not differ from WT. Asterisks refer to statistically significant differences: *P < 0.05, **P < 0.01, ***P < 0.001. Scale bars: 100 μm, IDD, iron deficient diet; IED, iron enriched diet; PVN, periventricular hypothalamic nucleus; mPFC, medial prefrontal cortex; BLA, basolateral amygdala; error bars, standard error of the mean. Statistically significant results are reported in Table 1S.

The marked anxious behaviour in *Tfr2-*KO mice suggests that *Tfr2* deletion in combination with iron overload might cause an abnormal activation of the anxiety system. We therefore investigated the expression pattern of cFos and Zif-268, the immediate early genes frequently used as markers for neuronal activity^47^, in brain nuclei belonging to the anxiety circuitry, including the hippocampus, the medial prefrontal cortex (mPFC), the basolateral (BLA), and central (CeA) amygdala and the hypothalamic paraventricular nuclei (PVN). Interestingly, in the hippocampus of *Tfr2*-KO mice the two activity markers were highly upregulated in CA3 (Fig. 5A,B,L,P) and CA1 neurons (Fig. 5A,B,F,G,I,J,M,Q), while their expression in the dentate gyrus (DG) did not differ from that of WT mice (Fig. 3SB and not shown). Of note, in *Tfr2*-KO mice fed with IDD anti-cFos and Zif-268 staining decreased to the levels of the WT mice in both CA3 and CA1 subregions (Fig. 5C,H,K,L,M,P,Q). The medial prefrontal cortex (mPFC) is one of the main targets of the hippocampal neurons and contributes to the anxiety control and stress responses by projecting to the BLA and, indirectly, to the PVN^39^. The levels of both cFos and Zif-268 increased significantly in this area of *Tfr2*-KO mice compared to WT or *Tfr2*-KO IDD mice (Fig. 5N,R). Consistently, both transcription factors appeared significantly upregulated in the BLA of the *Tfr2*-KO mice (Fig. 5O,S). Furthermore, anti-cFos/Zif-268 immunostainings did not reveal differences in activity levels of neurons included either in the PVN (Fig. 5D,E) and CeA (or in other areas unrelated to anxiety control) (not shown). In line with the maintenance of WT activation levels in the CeA and PVN, the corticotropin-releasing factor (CRF) immunostaining in the PVN of *Tfr2*-KO mice did not differ from that of WT mice (Fig. 4SA,B), showing that, despite being associated with a pronounced anxious behaviour, *Tfr2* deletion does not alter CRF release into the hypothalamo-hypophyseal portal system^38^. Accordingly, we did not find differences in corticosterone levels in *Tfr2*-KO serum compared to WT (Fig. 4SC). We further tested whether the altered activity pattern of *Tfr2*-KO was associated with changes in levels of BDNF, a key-regulator of synaptic plasticity and hippocampal activity, whose alterations were associated with iron changes and anxiety^48^. However, we did not find changes of BDNF mRNA levels in hippocampus of *Tfr2*-KO mice compared to WT animals (Fig. 4SD). Of note, IED in WT animals also triggered a response that promoted a diffuse and aspecific upregulation of cFos throughout most brain areas (Fig. 3S). Thus, iron alterations due to Tfr2 deficiency positively and specifically modulate neuronal activation in the CA3-CA1-mPFC-BLA circuitry, while they do not alter the neuroendocrine compartment implicated in anxiety regulation.

Given the high levels of expression of Tfr2 alpha in the Mossy fiber pathway, we further hypothesized that the activation of the anxiety circuitry were triggered by altered signals conveyed by Mf to CA3 neurons. Therefore, we investigated possible changes in the density of glutamatergic vGlut1/2+ terminals in CA3. While no difference was observed in the number of VGlut1+ puncta, the density of anti-VGlut2 positivity significantly increased at this site, suggesting incremented excitation and terminal remodelling (Fig. 5T,U,V). Collectively, these data are consistent with a role of Tfr2 alpha in the regulation of both the Mf output and the activity of the anxiety system.

### Increased microglia reactivity, dystrophic changes and death in Tfr2-KO mice

**Figure 6:**
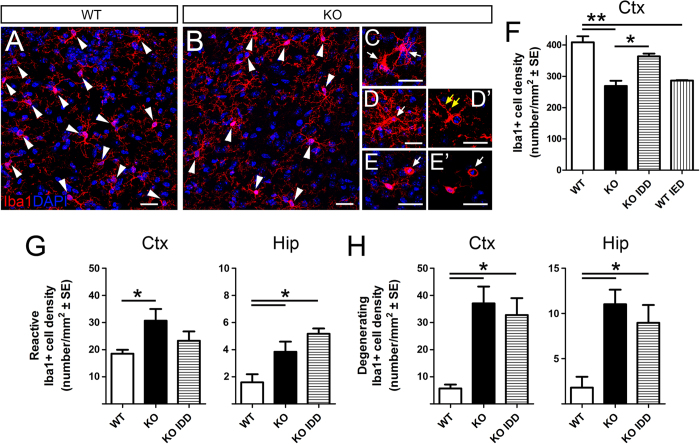
Microglial phenotypes in Tfr2-KO and WT mice. Immunofluorescence (**A**–E’) and quantification (**F**) of microglial cells using Iba1 marker in total brain. Quantification of reactive (**G**) and degenerating (**H**) cells in cortex (Ctx) and hippocampus (Hip). Arrowheads indicate Iba1+ microglia density (**A**,**B**). Arrows indicate reactive morphologies (**C**), signs of degeneration (yellow arrows, D’) and pyknosis (arrows in (**E**), E’). Asterisks refer to statistically significant differences: *P < 0.05, **P < 0.01. Statistically significant results are reported in Table 1S. CTX, neocortex (primary M1 motor cortex); HIP, hippocampus; IDD, iron deficient diet; IED, iron enriched diet; error bars, standard error of the mean. D’, E’, single optical slices. Scale bars: 20 μm.

Recent findings indicate that microglia alterations are frequently associated with increased stress and anxiety^49^. Although immunohistological analyses did not reveal alterations of the gross anatomy of the *Tfr2*-KO mouse brain (Fig. 5S), in *Tfr2*-KO we found a decrease in the density of microglial cells identified by labelling for the ionized calcium-binding adaptor molecule 1 (Iba1), (Fig. 6A,B). This decrease occurred throughout the brain and was quantified in *Tfr2*-KO mouse cerebral cortex (Fig. 6A,B,F) and hippocampus (Fig. 6G,H).

Here, the density of both reactive (i.e showing hypertrophy and very thick short processes Fig. 6C) or degenerating (i.e. bearing fragmented or dystrophic processes and a pyknotic nucleus; Fig. 6D–E’) Iba1+ cells appeared significantly increased compared to WT mice (Fig. 6G,H), suggesting that reactive and degenerative events occur in parallel and that the latter changes dominate, thereby resulting in the reduction of the microglial pool in the *Tfr2*-KO mice.

In order to understand the correlation of the *Tfr2*-KO microglial phenotype with iron overload and/or anxiety, we looked at microglia in *Tfr2-KO* IDD and WT IED mice. Despite recovering a physiological density of Iba1+ cells in the cerebral cortex, (Fig. 6F), KO IDD mice still displayed some microglia activation and degeneration in the examined areas (Fig. 6G,H). Low iron levels in these mice (Table 1) indicated that such microglial alterations might not be due to iron overload *per se.* Yet, the decrease in microglial density could be a factor participating in the behavioural abnormalities found in *Tfr2*-KO mice. However, in WT IED animals microglia are also diminished in the absence of anxiety signs (Fig. 6F). Moreover, microglial reactivity and degeneration occurred in both *Tfr2*-KO IDD (Fig. 5) and WT IED mice (Fig. 6S) in the absence of an anxious phenotype, indicating that these features are also not directly linked to anxiety. Interestingly, we found signs of ongoing inflammation (as monitored by levels of the Serum Amyloid A1 (SAA1) acute phase protein^50^; Fig. 7S) in the brain of WT IED mice but not in those of *Tfr2*-KO animals, suggesting that Tfr2 may be implicated in the regulation of the inflammatory profile of these cells. Thus, microglia appear to strongly respond to alteration of iron metabolism but they do not play a specific role in the behavioural alterations of *Tfr2*-KO.

## Discussion

In this work we show that *Tfr2* germinal silencing affects brain iron homeostasis. Furthermore, we reveal that Tfr2 alpha is highly expressed in neurites of brain circuits of anxiety and stress disorders. This pattern, together with the prominent anxious behaviour of *Tfr2*-KO mice, strongly suggests a role for Tfr2 alpha in the regulation of anxiety circuits. Finally, our results further highlight a particular sensitivity of microglia to perturbations of iron metabolism, even when peripheral iron accumulation is moderate and does not associate with behavioural alterations.

Tfr2 alpha is the mainly transcribed isoform of the *TFR2* gene, whose mutations are responsible of a form of hereditary hemochromatosis named HFE3^6^. Hereditary haemochromatosis is a genetically heterogeneous disease due to functional impairment of the iron hormone Hepc and of several Hepc regulating proteins^3^.

Tfr2 alpha is a key iron sensor that, in liver, triggers a signal transduction cascade that activates the expression of Hepc, a small protein that reduces iron efflux from cells and leads to its intracellular accumulation^2^. Tfr2 beta isoform is instead known to exert a role in iron efflux in spleen reticuloendothelial cells^9^. So far, several papers reported a Tfr2 alpha expression in the nervous tissue^23^,^24^,^25^ that was proposed to be restricted to specific brain regions^23^,^24^. Our transcriptional and immunohistological analyses validated in *Tfr2*-KO mice, showed relevant Tfr2 alpha expression in the nervous tissue and revealed Tfr2 alpha protein distribution in the neurite compartment of limbic areas implicated in anxiety and stress response. This expression pattern is not fully consistent with those previously reported in human tissues^23^, that described Tfr2 expression only in the cerebellum. While this discrepancy may be due to species-specific factors, open access transcriptome data published on GEO Profiles indicate that in both humans and rodents Tfr2 is not exclusively expressed in the cerebellum and is detected in hippocampus, cerebral cortex, basal ganglia and amygdala (Profile: GDS2678/40311_at/, Brain regions of humans and chimpanzees; Profile: GDS1406/160674_at/Tfr2, Brain regions of various inbred strains). Further, a low and ubiquitous Tfr2 alpha expression in mouse neural cells and brain endothelium below the sensitivity of the detection approach applied in this study may occur. Indeed, the diffuse upregulation of Hepc in WT IED brains supports the presence of a more widespread Tfr2 alpha expression.

Based on the key role of Tfr2 in regulating liver iron load, one obvious expectation was to find an increased iron amount in the parenchymal nervous tissue upon Tfr2 deletion. Indeed, in total brain extracts of *Tfr2*-KO including circulating blood, BIC was significantly higher than that of both WT and WT IED, underscoring that in these animals brain parenchyma is exposed to a higher iron amount. Moreover, Perls’ staining on brains from *Tfr2*-KO perfused mice revealed that iron accumulates in some of the brain regions where we found relevant Tfr2 expression (hippocampal CA1 and CA3, PVN) as well as in compartments, such as the choroid plexi, which are sites of iron trafficking between systemic circulation and the brain environment^51^.

Yet, despite iron deposition, no changes in Hepc protein levels were observed in the nervous tissue, as detected by WB and immunofluorescence. Thus, the strong Hepc reduction in samples containing circulating blood reflects essentially peripheral/systemic Hepc blunting in the absence of Tfr2.

In line with an increased iron amount in *Tfr2*-KO brain, levels of the iron storage protein ferritin globally increase. Ferritin H subunit in particular, is specifically increased in *Tfr2*-KO mice. This induction may reflect the need to counteract the deleterious effects of an enhanced iron-based Fenton reaction, which produces damaging hydroxyl radicals^52^. Tf protein is overexpressed in both WT IDD and *Tfr2-*KO IDD brain, as expected in condition of iron deprivation^44^,^45^. Unexpectedly Tf displays an increase, although not statistically significant, also in *Tfr2*-KO brains from non-perfused mice, while it is comparable to WT levels in brains from perfused animals. These results are consistent with the additional presence of blood Tf in non-perfused brains.

There is a tendency to decrease for the iron importer DMT1 in *Tfr2*-KO brains, in agreement with a higher iron content, at least in defined cell type(s) of the brain tissue. Moreover, its increase both in *Tfr2*-KO IDD and in WT IDD mice brain is a correct response to iron deprivation based on the activation of the intracellular iron regulatory IRE/IRP response system^45^. Overall these alterations are consistent with a Tfr2 alpha-dependent dysfunctional iron handling in the brain, even though we cannot exclude that over time an exacerbation of the iron burden in WT IED animals could eventually lead to a phenotype overlapping that of *Tfr2*-KO mice.

From the behavioural point of view, iron increase in *Tfr2*-KO mice was accompanied by anxious-like behaviour as assessed by the EPM, where *Tfr2*-KO SD mice spent proportionally less time in the anxiogenic arm compared to other genotypes and conditions. Anxiety-like behaviours were dependent on iron increase, because they were reverted by IDD in *Tfr2*-KO mice. Despite in this study we cannot definitively dissect the contribution of systemic vs. parenchymal iron overload to the anxious phenotype, lack of anxiety signs in WT IED mice that are systemically overloaded but with normal brain iron content - as notably detected without perfusion to wash away the contaminating blood-, suggests that increased parenchymal iron deposits play a prominent role in promoting behavioural alterations. However, the lower systemic iron load in WT IED mice compared to *Tfr2*-KO mice may take part in the absence of the phenotype. In former studies both iron deficiency and iron overload during critical developmental windows or at adult ages have been shown to affect emotional behaviour in rodents^53^. However, the biological mechanisms mediating the effect of iron level alterations at early or mature stages are likely to be distinct. While precocious actions are plausible to rely on abnormal circuit formation, here we find changes of circuit activity, possibly accompanied by some degree of remodelling (see below). In line with our findings, a former study on the effects of experimental brain iron overload (i.p. injections of 3 mg/kg of ferrous sulphate for 5 consecutive days) also reported anxiety in rats, though in association with defects in spatial learning^54^. Moreover, iron-deficiency was also shown to lead to anxiety in mutant mice, likely due to the iron requirement in the synthesis of serotonin and noradrenaline^48^. Since WT IDD mice in this study did not show a brain-specific iron decrease, they are not expected to show behavioural alterations associated with brain iron deficiency. Finally, no data are known about brain iron metabolism in the few HFE3 iron overloaded patients available and no sign of neurological alterations has been evidenced so far. Nevertheless, it must be taken into account that these patients nowadays undergo early diagnosis and efficient phlebotomy until serum iron parameters normalization.

Consistent with behavioural data, in *Tfr2*-KO mice we found a specific and selective overactivation of the limbic circuits controlling anxiety and stress responses, as demonstrated by increased expression of cFos and Zif-268 (Fig. 8S). Surprisingly, also IED in WT mice promoted cFos/Zif-268 upregulation in the brain, but that was far more broad and intense compared to *Tfr2*-KO animals, suggesting that distinct mechanisms account for the induction in the two experimental models. These data provide in-depth explanation to former hints indicating that alterations of iron homeostasis affect expression of neurotransmitters and trophic factors^15^,^16^,^26^,^48^,^53^. Importantly, in *Tfr2*-KO cFos/Zif-268 upregulation declined in IDD, thereby showing dependence on iron levels. Notably, in *Tfr2*-KO mice all the limbic stations displayed enhanced activity, with the exception of areas belonging to the neuroendocrine stress axis, whose functioning appears unaffected as shown by absence of alterations in PVN CRF and blood corticosterone (Fig. 4S). Moreover, we report an increased V-Glut2 positivity compared to control levels in the CA3 Mf terminal field suggesting that Tfr2 deletion may affect the final output of the Mf pathway by inducing terminal remodeling or promoting immature traits in these fibers, in line with restriction of V-Glut2 during immature developmental stages^55^. Several studies have shown a positive correlation between Mf sprouting and increased anxiety-like behaviour in rodents^56^,^57^,^58^,^59^, suggesting that the Mf system may contribute to modulation of anxiety-like responses. Thus, collectively data are consistent with a model where Tfr2-dependent alterations in iron homeostasis affect the activity of the main brain areas responsible for the neural control of emotional behaviour, and promote anxiety. Further, high Tfr2 alpha expression along Mf and in nuclei of the limbic circuit suggests a specific role for this isoform in the regulation of the anxiety circuits.

It is interesting to note that activity alterations and behavioural abnormalities in *Tfr2*-KO mice are not due to degenerative events in neurons or astrocytes. We found degeneration only in microglia, that also display increased iron storage in KO mice, as detected by Perls’ staining. This suggests that iron-mediated challenge may be compensated in other neural cells. Yet, microglial loss and alterations were also found in WT IED brains that did not show behavioural alterations, ruling out their role in the detected anxious-like traits. Nevertheless, microglial cells were clearly affected in the *Tfr2*-KO mice and displayed reactivity, dystrophic changes and death. These findings are in line with their known action as buffering elements that counteract disturbances in iron regulation^60^. However, in our study, microglial alterations are detected not only in *Tfr2*-KO SD but also in WT IED mice, thereby excluding their specific dependence on Tfr2 abrogation and supporting the hypothesis that either the altered or increased iron processing is responsible for the observed dystrophic modifications and degeneration in microglia.

Taken together, these data add to the growing body of evidence that alterations of systemic iron loading affect brain homeostasis and functioning, and reveal a specific role for *Tfr2*-dependent iron overload in the control of iron regulatory network in the brain tissue as well as in the control of anxious behaviours.

## Additional Information

**How to cite this article**: Pellegrino, R. M. *et al.* Transferrin Receptor 2 Dependent Alterations of Brain Iron Metabolism Affect Anxiety Circuits in the Mouse. *Sci. Rep.* **6**, 30725; doi: 10.1038/srep30725 (2016).

## Supplementary Material

Supplementary Information

## Figures and Tables

**Table 1:** Brain and liver iron amount and hepatic Hepc transcription.

Genotype Diet	WT SD	WT IDD	WT IED	***Tfr2***-KO SD	***Tfr2-***KO IDD
BIC *(μg/g dry tissue)*	237.3 ± 30.8	189.8 ± 17.4**°°°**	240.2 ± 30.5**°**	317.8 ± 81.9**	175.7 ± 25.3*, **°°°**
BIC^ *(μg/g dry tissue)*	154.5 ± 18.3	ND	ND	178.1 ± 21.7	ND
LIC *(μg/g dry tissue)*	480.2 ± 116.7	246.1 ± 34.5°°°	1099.2 ± 87.2°	1839.3 ± 448.9***	326.2 ± 105.4**°°°**
hepatic Hepc *(ΔΔCt mean)*	1 ± 0.152**°°**	0.003 ± 0.001***	2.78 ± 0.360***, **°°°**	0.424 ± 0.174**	0.002 ± 0.003***

WT, wild type; SD, standard diet; IDD, iron deficient diet; IED, iron enriched diet; ND, not determined; ^, perfused brain. * significance vs WT; ° significance vs *Tfr2*-KO.

Only statistically significant results vs WT and Tfr2 KO are shown. All the other statistically significant results are reported in Table 1S.
