# Supplementary material for: Transferrin Receptor 2 Dependent Alterations of Brain Iron Metabolism Affect Anxiety Circuits in the Mouse
Source: Sci Rep. 2016 Aug 1;6:30725. doi: 10.1038/srep30725 (PMC4967901; doi:10.1038/srep30725)
Supplement: Supplementary Information [file srep30725-s1.pdf]

TRANSFERRIN RECEPTOR 2 DEPENDENT ALTERATIONS OF BRAIN IRON METABOLISM AFFECT ANXIETY CIRCUITS IN THE MOUSE

Rosa Maria Pellegrino, Enrica Boda, Francesca Montarolo, Martina Boero, Mariarosa Mezzanotte, Giuseppe Saglio, Annalisa Buffo and Antonella Roetto

Supplemental Experimental Procedure

Molecular biology analyses

The primers used SYBR Green PCR technology are listed below:

mTfr2 alpha FW 5'GCCATGTTTCTCCCGGTTCCT3'

mTfr2 beta FW 5'CCTGGCCCCTAGTGTGATTTC3'

mTfr2REV 5'TGGCGCGAGAGCTTATCG3';

mBDNF III FW 5'GCTTTCTATCATCCCTCCCCGAGAGT3'

mBDNFREV 5'GAAGTGTACAAGTCCGCGTCCTTA3' (Aid et al., 2007).

Behavioral tests

Spatial learning and memory of mice were investigated by means of *Morris water maze* test. The maze was located in a room with numerous extra-maze cues visible to the mice during testing. It consisted of a circular pool (140 cm diameter; 40 cm height) filled with water at 25° C to avoid hypothermia added with milk powder to hide a submerged platform. A small escape platform made of transparent plastic (15 cm diameter) was placed in the centre of one quadrant 35 cm far from the side-wall of the pool at a fixed position and was hidden 1 cm beneath the water surface. The acquisition phase (days 1-4) consisted of 4 training days (days 1-4) with four trials per day with a 15 minutes inter-trial interval. Four points equally spaced along the circumference of the pool (i.e. north, south, west, east) served as the starting position, which was randomized across the four trials each day. For each trial, the mouse was released from the side of the pool, facing the wall. The time spent to reach platform (escape latency) was recorded for each animal. If an animal did not reach the

platform within 90 seconds, it was guided to the platform, where it was allowed to rest for 30 seconds, before being returned to the home cage. Between trials mice were kept dry in a cage filled with paper towels .During the first trial of the first day (when the mice knew anything about the spatial location of the platform) the dis tance travelled and the time spent in centre zone of the pool (70 cm diameter) were calculated and expressed as percentages. On day 5, a probe trial was performed to assess spatial memory after a 24 hours delay since the last acquisition trial. In the probe trial, the platform was removed from the maze and the animals were allowed to swim freely for 60 seconds. The time spent in each quadrant (quadrant 1, Q^1; quadrant 2, Q^2; quadrant 3, Q^3 and target quadrant, Q^T, in which the escape platform was located during the acquisition phase) was recorded. The primary retention measure was the accuracy ratio (AR)[34] calculated as follows: time spent in the target quadrant (TQ^T) multiplied by 3 and divided by the time spent in the other three quadrants ($TQ^1 + TQ^2 + TQ^3$). An AR of 1 corresponds to chance level, indicating no preference for Q^T relative to the other quadrants. An AR of 2 corresponds to 24 s spent in Q^T, while an accuracy ratio of 3 corresponds to 30 s in Q^T. In the probe trial, also path length before and after the target zone was reached, swim velocity and distance were assessed. Data were analyzed in the digitized image using a computerized video-tracking software (Ethovision XT video track system; Noldus Information Technology, Wageningen, Netherlands).

Tfr2-KO and WT mice in SD, IDD and IED were also tested for *elevated plus maze* (EPM) test to detect anxious-like behaviors. The EPM test apparatus was a plus-cross shaped constructed from gray forex raised 60 cm above the floor. It comprised two open arms (30 cm x 5 cm x 0.20 cm) and two closed arms (30 cm x 5 cm x 15 cm walls) originating from a central platform (5 cm x 5 cm). On test day, mice were transported to a dimly illuminated (2 x 40W, indirect) testing room, adjacent to the animals' room, and left undisturbed for at least 1 h

before testing. At the beginning of each trial each animal was gently placed in the centre of the plus maze, facing an open arm and it was allowed to explore the maze for 5 min. At the end of each trial, the apparatus was accurately cleaned up with ethanol 2% and water. Data were recorded automatically as described above. The cumulative time spent in either open or closed arms and the respective number of entries was recorded. An animal was considered to have entered an arm of the plus maze when all four paws had left the central platform.

Table 1S

Figure/Table	Applied Test	P value	F value	Post hoc analyses	Post hoc results
1B	One-way Anova	0.0283	(2,14)=4.684	Bonferroni's Multiple Comparison Test	WT IDD vs. WT IED, P<0.05
3A	One-way Anova	0.0002	(4,10)=17.53	Bonferroni's Multiple Comparison Test	WT vs. TFR2-KO, P<0.01<br>TFR2-KO vs. WT IED, P<0.001<br>WT IDD vs. WT IED, P<0.01<br>WT IED vs. TFR2-KO IDD, P<0.01
3N	One-way Anova	0.0002	(4,38)=7.417	Bonferroni's Multiple Comparison Test	WT vs. WT IED, P<0.05<br>WT vs. TFR2-KO, P<0.001<br>TFR2-KO vs. WT IDD, P<0.05<br>TFR2-KO vs. TFR2-KO IDD, P<0.05
3O	One-way Anova	0.002	(4,9)=10.34	Bonferroni's Multiple Comparison Test	WT vs. TFR2-KO IDD, P<0.01<br>WT IED vs. TFR2-KO IDD, P<0.01
3P	One-way Anova	0.3303	(4,8)=1.355		
3Q	One-way Anova	<0.0001	(4,22)=9.905	Bonferroni's Multiple Comparison Test	WT vs. WT IDD, P<0.01<br>TFR2-KO vs.WT IDD, P<0.001<br>WT IDD vs WT IED, P<0.05<br>WT IDD vs TFR2-KO IDD, P<0.05
4A	Two-way Repeated-Measures Anova	Genotype effect, P=0.29; Time effect, P<0.0001	(3,45)=52.16	Bonferroni's Multiple Comparison Test	
4B	Mann-Whitney U test	0.23			
4C	Mann-Whitney U test	0.04			
4D	Mann-Whitney U test	n.s.			
4E	One-way Anova	<0.0001	(4,78)=7.95	Bonferroni's Multiple Comparison Test	WT vs. TFR2-KO, P<0.001<br>TFR2-KO vs. WT IDD, P<0.001
4F	One-way Anova	0.0003	(4,78)=6.08	Bonferroni's Multiple Comparison Test	WT vs. TFR2-KO, P<0.001<br>TFR2-KO vs. WT IDD, P<0.05
4G	One-way Anova	0.0002	(4,78)=6,27	Bonferroni's Multiple Comparison Test	WT vs. TFR2-KO, P<0.001
5L	One-way Anova	0.024	(2,10)=5.58	Bonferroni's Multiple Comparison Test	WT vs. TFR2-KO, P<0.05<br>TFR2-KO vs. TFR2-KO IDD, P<0.05
5M	One-way Anova	0.0056	(2,7)=11.93	Bonferroni's Multiple Comparison Test	WT vs. TFR2-KO, P<0.01<br>TFR2-KO vs. TFR2-KO IDD, P<0.05
5N	One-way Anova	0.0004	(2,15)=13.57	Bonferroni's Multiple Comparison Test	WT vs. TFR2-KO, P<0.01<br>TFR2-KO vs. TFR2-KO IDD, P<0.001
5O	One-way Anova	0.0184	(2,7)=7.46	Bonferroni's Multiple	WT vs. TFR2-KO, P<0.05

				Comparison Test	TFR2-KO vs. TFR2-KO IDD, P<0.05
5P	One-way Anova	0.0309	(2,8)=6.5	Bonferroni's Multiple Comparison Test	WT vs. TFR2-KO, P<0.05<br>TFR2-KO vs. TFR2-KO IDD, P<0.05
5Q	One-way Anova	<0.0001	(2,8)=51.12	Bonferroni's Multiple Comparison Test	WT vs. TFR2-KO, P<0.01<br>TFR2-KO vs. TFR2-KO IDD, P<0.001
5R	One-way Anova	<0.0001	(2,7)=17.10	Bonferroni's Multiple Comparison Test	WT vs. TFR2-KO, P<0.01<br>TFR2-KO vs. TFR2-KO IDD, P<0.001
5S	One-way Anova	0.0193	(2,7)=7.317	Bonferroni's Multiple Comparison Test	WT vs. TFR2-KO, P<0.05
5T vGlut1	Unpaired t test	n.s.			
5T vGlut2	Unpaired t test	0.0068			
6F	One-way Anova	0.0002	(3,10)=18.87	Bonferroni's Multiple Comparison Test	WT vs. TFR2-KO, P<0.01<br>TFR2-KO vs. TFR2-KO IDD, P<0.05<br>WT vs. WT IED, P<0.01
6G Ctx	One-way Anova	0.0416	(2,6)=7.515	Bonferroni's Multiple Comparison Test	WT vs. TFR2-KO, P<0.05
6G Hip	One-way Anova	0.0144	(2,6)=9.328	Bonferroni's Multiple Comparison Test	WT vs. TFR2-KO, P<0.05
6H Ctx	One-way Anova	0.0099	(2,6)=10.95	Bonferroni's Multiple Comparison Test	WT vs. TFR2-KO, P<0.05
6H Hip	One-way Anova	0.0159	(2,6)=8.923	Bonferroni's Multiple Comparison Test	WT vs. TFR2-KO, P<0.05
Table 1 (BIC)	One-way Anova	<0.0001	(4,30)=11.8	Bonferroni's Multiple Comparison Test	WT vs. TFR2-KO, P<0.01<br>WT vs. TFR2-KO IDD, P<0.05<br>TFR2-KO vs. WT IDD, P<0.001<br>TFR2-KO vs. WT IED, P<0.05<br>TFR2-KO vs. TFR2-KO IDD, P<0.001
Table 1 (LIC)	One-way Anova	<0.0001	(4,16)=41.23	Bonferroni's Multiple Comparison Test	WT vs. TFR2-KO, P<0.001<br>TFR2-KO vs. WT IDD, P<0.001<br>TFR2-KO vs. WT IED, P<0.05<br>TFR2-KO vs. TFR2-KO IDD, P<0.001<br>WT IDD vs. WT IED, P<0.01<br>WT IED vs TFR2-KO IDD, P<0.01
Table 1 (hepatic Hepc)	One-way Anova	<0.0001	(4,10)=114	Bonferroni's Multiple Comparison Test	WT vs. WT IDD, P<0.001<br>WT vs WT IED, P<0.001<br>WT vs TFR2-KO, P<0.01<br>WT vs. TFR2-KO IDD, P<0.001<br>WT IED vs. TFR2-KO, P<0.001<br>WT IDD vs. WT IED, P<0.001

					WT IED vs. TFR2-KO IDD, P<0.001
Suppl. 1 C	Unpaired t test	0.0142			
Suppl. 2A	One-way Anova	0.0005	(2,9)=19.89	Bonferroni's Multiple Comparison Test	WT vs. WT IED, P<0.01
Suppl. 2B	One-way Anova	0.0030	(2,10)=10,93	Bonferroni's Multiple Comparison Test	WT vs. WT IED, P<0.01
Suppl. 2C	One-way Anova	0.0036	(2,8)=12.35	Bonferroni's Multiple Comparison Test	WT vs. TFR2-KO, P<0.01<br>WT vs. WT IED, P<0.01
Suppl. 5A Ctx	Unpaired t test	0.0005			
Suppl. 5A Hip	Unpaired t test	0.0258			
Suppl. 5B Ctx	Unpaired t test	0.0021			
Suppl. 5B Hip	Unpaired t test	0.0045			

Table 1S.

Details of performed statistical analyses. WT, wild type; SD, standard diet; IED, iron enriched diet; IDD, iron deficient diet. Only comparisons leading to P<0.05 are reported.

Supplementary Figures

Figure 1S

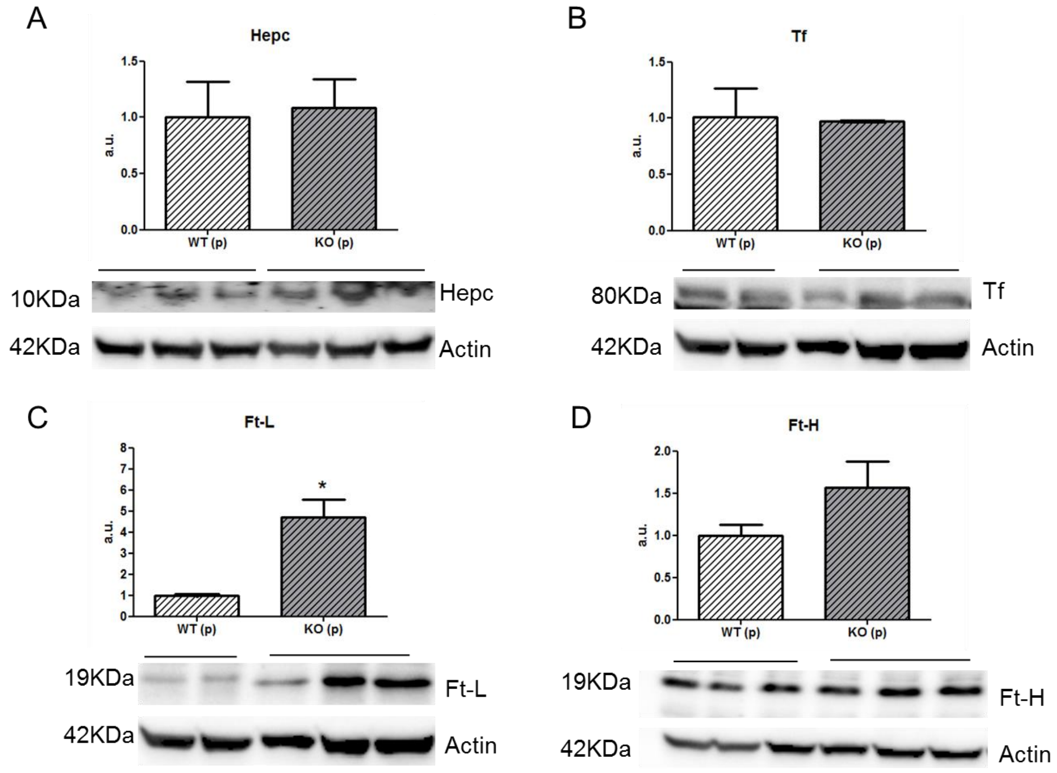

Figure 1S. Iron protein quantification in brain of perfused mice.

Western blot analysis and quantification of brain Hepcidin (Hepc) (A), Transferrin (Tf) (B) and L-H Ferritins (Ft-L, Ft-H) (C,D) proteins. Results are shown as averages ± standard error of the mean. Symbols refer to a statistically significant difference: *P<0.05. WT, wild type; KO, *Tfr2*-KO; a.u., arbitrary unit; (p), perfused.

Figure 2S

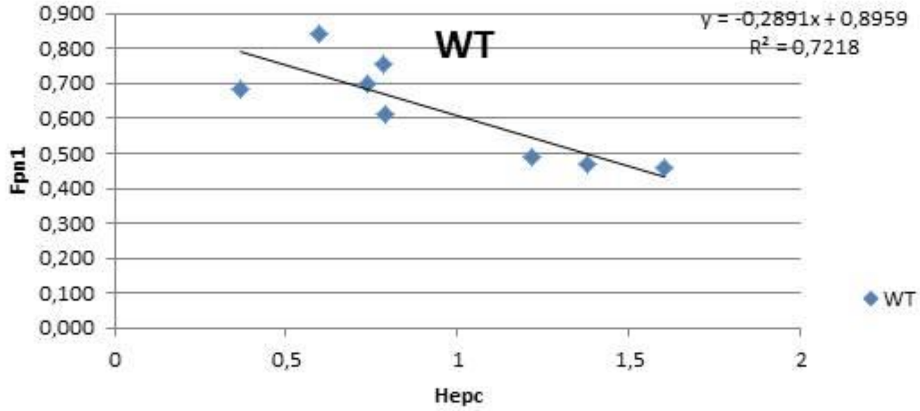

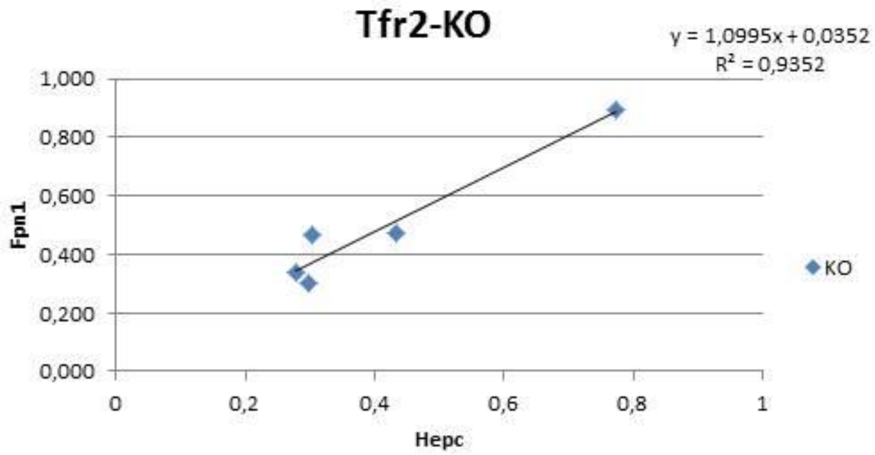

Figure 2S. Linear regression of Hepc-Fpn1 relationship

Linear regression between Hepcidin (Hepc) and Ferroportin (Fpn1) in WT and Tfr2-KO mice. Despite the different slope signs, 95% confidence interval shows an overlap of distributions of WT and KO data (- 0.110, 1.078 for WT; 0.278, 1.630 for Tfr2-KO). Values were obtained from Western blot analysis quantifications.

Figure 3S

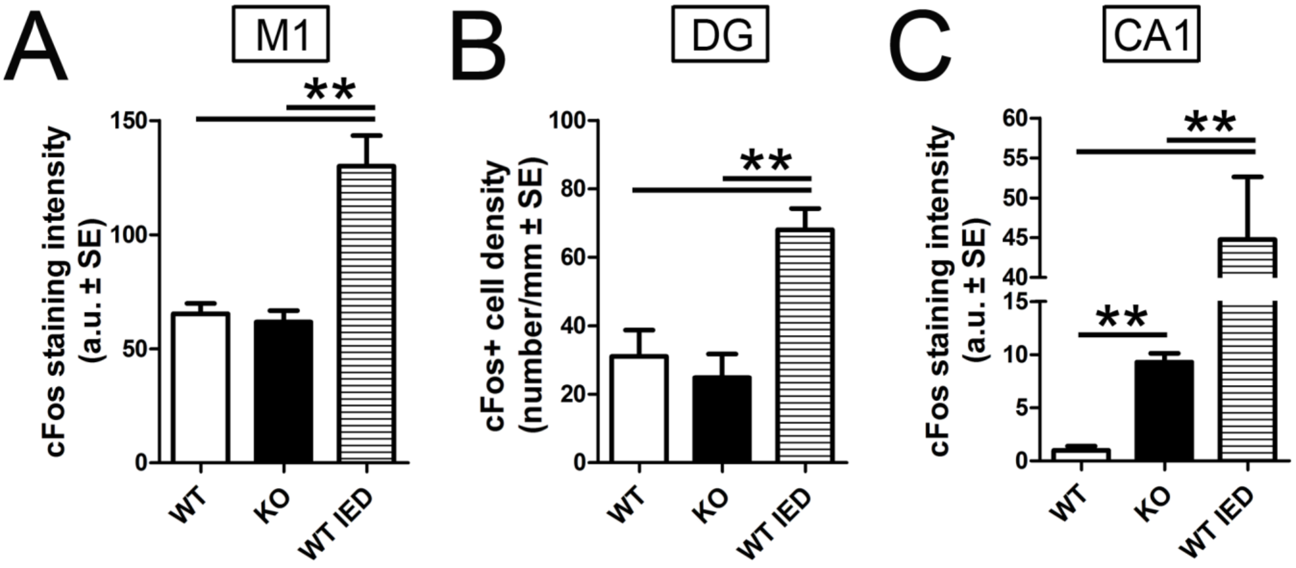

Figure 3S. Widespread and aspecific activation of neuronal circuitries in WT IED mice

Quantification of cFos+ nuclei shows activation of neuronal circuitries in the primary motor cortex (A), hippocampal dentate gyrus (B) and CA1 (C), indicating that IED does not specifically affect the activation state of the limbic circuit. One-way ANOVA, main effect of group A, $F(2,9)=19.89$, B, $F(2,10)=10.93$, C, $F(2,8)=12.35$. M1, primary motor cortex, DG, dentate gyrus, CA1, Cornus Ammonis 1. **$P<0.01$, ***$P<0.001$. Error bars, standard error of the mean. WT, wild type; KO, Tfr2-KO; IED iron enriched diet.

Figure. 4S

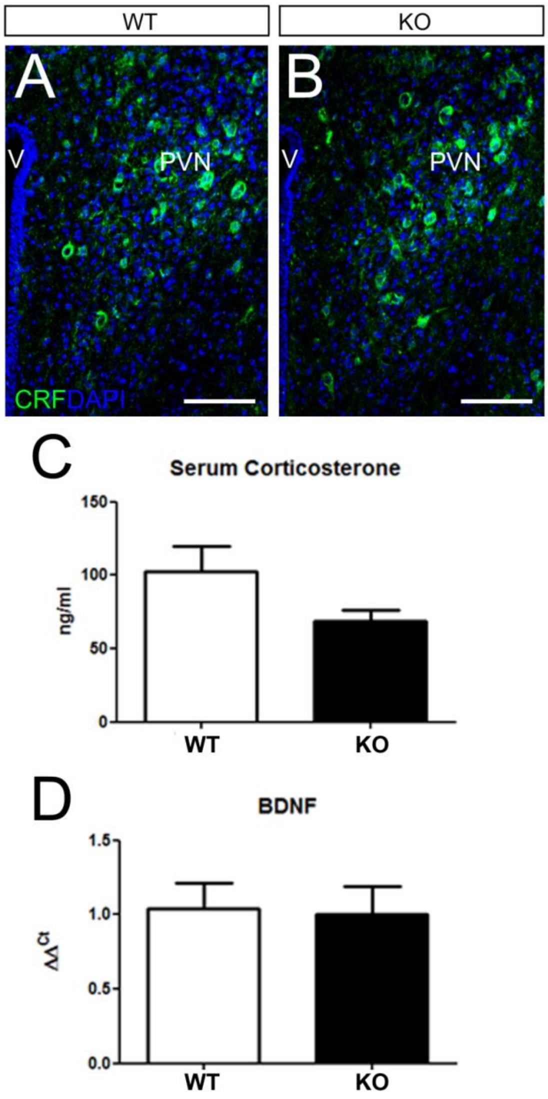

Figure 4S. Analysis of the corticotropin-releasing factor (CRF) in the PVN, corticosterone levels in serum and BDNF (Brain-derived neurotrophic factor) expression in hippocampus

(A,B) Expression of corticotropin releasing factor is unaltered in the PVN of Trf2-KO mice. (C) Corticosterone levels in Tfr2-KO serum are not different from those of WT mice. (D) BDNF

mRNA expression levels in Tfr2-KO hippocampus are not different compared to WT. Non-parametric Student *t* test (unpaired, 2 tailed) is performed. Results are shown as averages ± standard error of the mean. Scale bars: 100 μm, PVN periventricular hypothalamic nucleus; WT, wild type; KO, Tfr2-KO animals.

Figure 5S

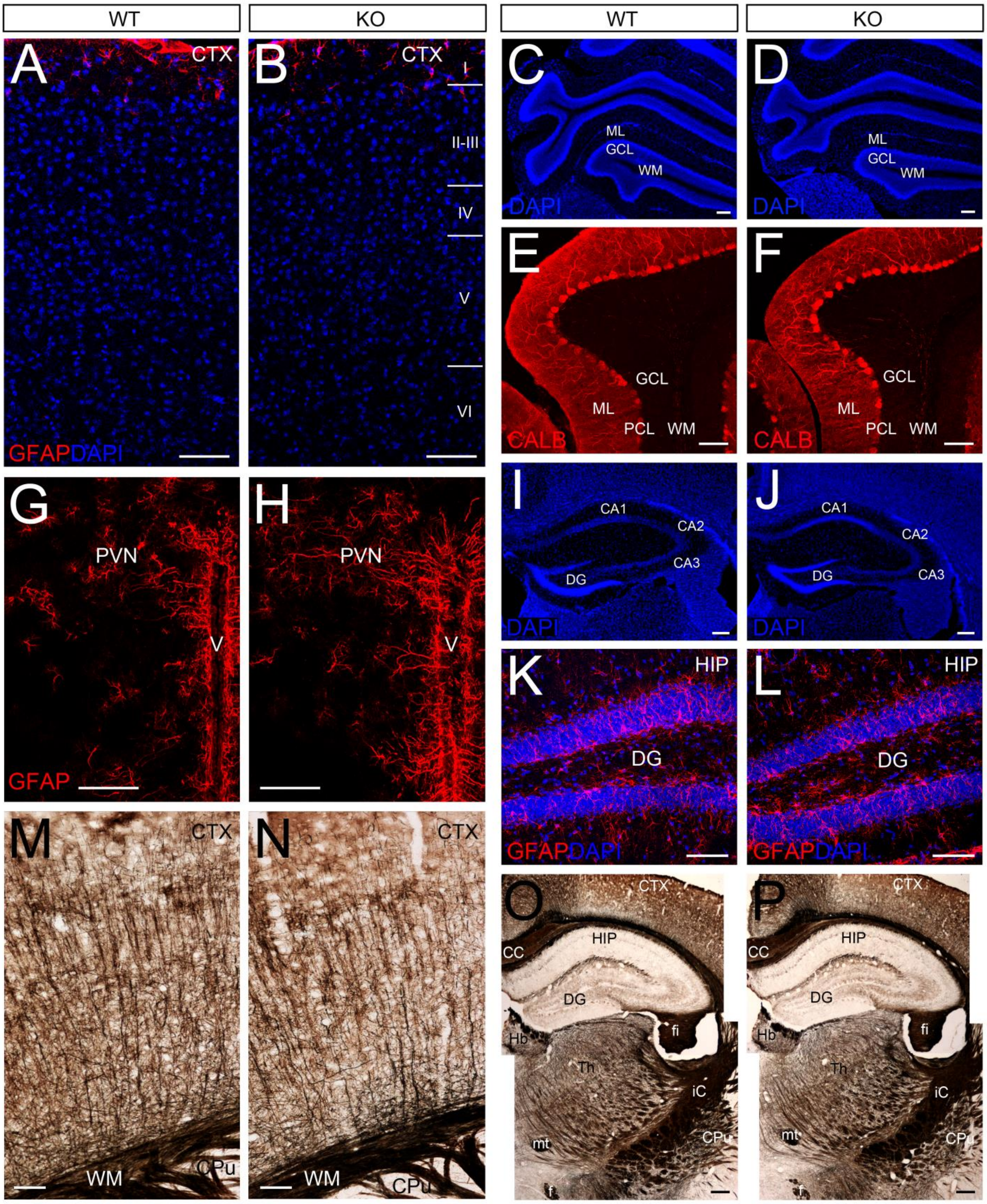

WT
KO
WT
KO
A
CTX
B
CTX
I
II-III
IV
V
VI
GFAPDAPI
C
ML
GCL
WM
DAPI
D
ML
GCL
WM
DAPI
E
GCL
ML
PCL
WM
CALB
F
GCL
ML
PCL
WM
CALB
G
PVN
V
GFAP
H
PVN
V
I
CA1
CA2
CA3
DG
DAPI
J
CA1
CA2
CA3
DG
DAPI
K
HIP
DG
GFAPDAPI
L
HIP
DG
GFAPDAPI
M
CTX
WM
CPu
N
CTX
WM
CPu
O
CTX
CC
HIP
DG
Hb
fi
Th
iC
mt
CPu
f
P
CTX
CC
HIP
DG
Hb
fi
Th
iC
mt
CPu
f

Figure 5S Gross brain anatomy in *Tfr2*-KO and WT mice

GFAP expression is not upregulated in the cerebral cortex (A,B) nor in the PVN (G,H) or hippocampus (K,L) of Tfr2-KO mice compared to age and sex-matched WT animals. No overt layering alterations are detected in the cerebral cortex (A,B), cerebellum (C,D) and hippocampus (I-J) of mutant mice compared to WT ones. Tfr2-KO calbindin-positive Purkinje neurons appear normal as regards cell morphology and number (E,F). No changes in myelination occur in either grey or white matter areas of Tfr2-KO mutants (M,N,O,P). Scale bars: 100 µm. WT, wild type, DG, dentate gyrus, HIP, hippocampus, CA1-2-3, Cornus Ammonis 1-2-3, CTX, neocortex (primary M1 motor cortex in A,B; somatosensory S1 cortex in M,N), CRB, cerebellum; PVN, paraventricular nucleus of the hypothalamus; WM, white matter; CPu, caudate-putamen; CRB, cerebellum, ML, molecular layer; GCL, granule cell layer; PCL, Purkinje cell layer; Hb, Habenula, Fi, hippocampal fimbria; F, fornix, Mt, mammillothalamic tract; iC, internal capsule; Th, thalamus, CC, corpus callosum; CALB, calbindin; GFAP, glial fibrillary acidic protein.

Figure 6S.

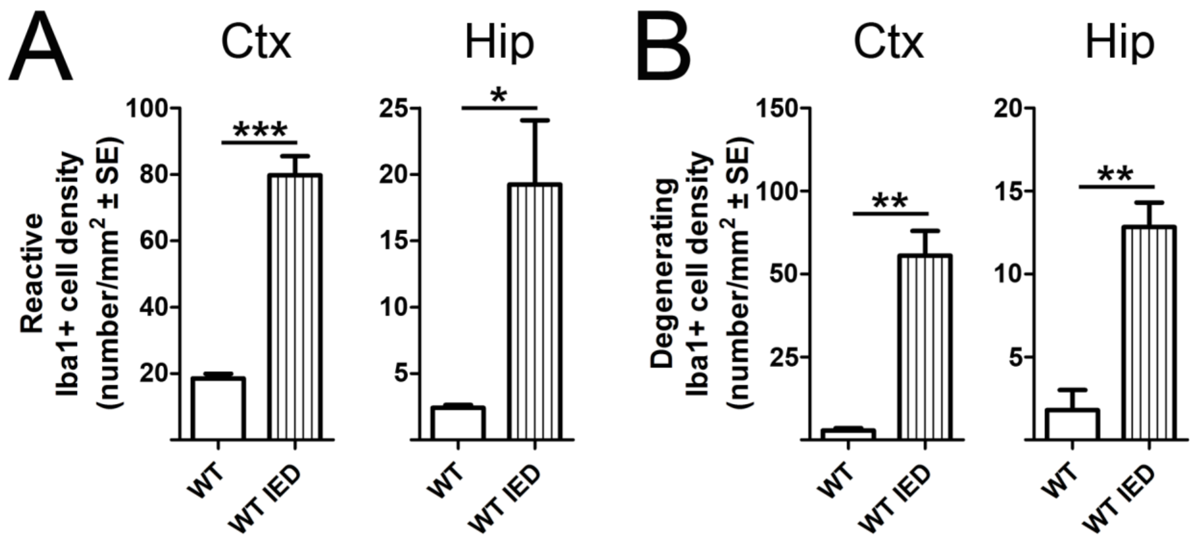

Figure 6S. Microglial phenotypes in WT mice upon iron-enriched diet (IED). Quantification of Iba1-positive reactive (A) and degenerating (B) cells in cortex (Ctx) and hippocampus (Hip). Asterisks refer to statistically significant differences: *P<0.05, **P<0.01, ***P<0.001. WT, wild type; IED, iron enriched diet; error bars, standard error of the mean.

Figure 7S

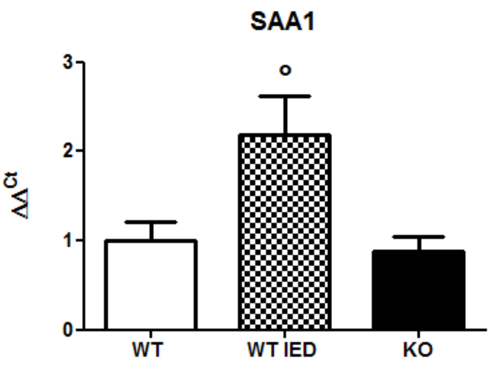

Figure 7S. SAA1 expression in the adult telencephalon

SAA1 transcription results to be increased in WT IED animals brain in comparison to Tfr2-KO mice. One-way ANOVA main effect of group P=0.0243, F (2.7)=6.627. Results are shown as averages ± standard error of the mean. Symbols refer to a statistically significant difference: °P<0.05 vs Tfr2-KO animals. WT, wild type; KO, Tfr2-KO; IED iron enriched diet

Figure 8S

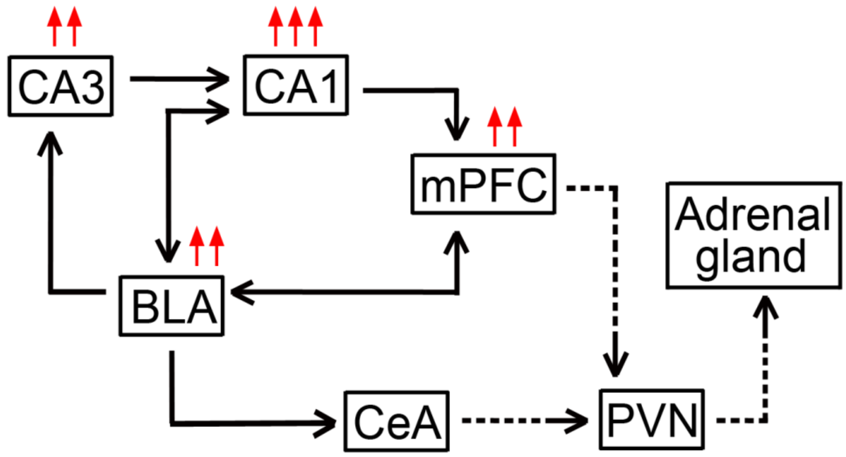

Figure 8S. Alteration of neuronal activity in the anxiety circuits

Diagram representing the anxiety circuits and the increment of neuronal activity at specific stations, as detected by immediate early genes activation. Neuroendocrine components are not implicated in the abnormal activation pattern found in *Tfr2*-KO mice. PVN, periventricular hypothalamic nucleus; mPFC, medial prefrontal cortex; BLA, basolateral amygdala; CeA, central nucleus of the amygdala; CA, Cornus Ammonis.
